# Supplementary material for: Collective memory for American leaders: Measuring recognition for the names and faces of the US presidents
Source: PLoS One. 2021 Jul 26;16(7):e0255209. doi: 10.1371/journal.pone.0255209 (PMC8313295; doi:10.1371/journal.pone.0255209)
Supplement: Attachment — Submitted filename: Putnam et al. Reviewer Response.docx [file pone.0255209.s001.docx]

Adam L. Putnam

Assistant Professor

Department of Psychology

Furman University

Greenville SC 29613

[adam.putnam@furman.edu](mailto:adam.putnam@furman.edu)

864.294.3213

June 25, 2021

Alexander N. 'Sasha' Sokolov, Ph.D.
Academic Editor
PLOS ONE

Dear Dr. Sokolov,

Thank you for the opportunity to address concerns related to online samples in the current set of studies. In response to your suggestion we added two paragraphs to the general discussion where we highlight potential concerns with using online samples (such as Amazon’s Mechanical Turk), and whether such concerns limit the generalizability of our results.

In addition, as requested by the editorial office we added a sentence to the method section clarifying that our studies had been approved by the Furman University IRB.

Sincerely,

Adam L. Putnam, PhD	Andy DeSoto, PhD

Assistant Professor of Psychology	Director of Government Relations

Furman University	Association for Psychological Science
